# Effects of Al-Ti-C Refiner and Forming Processes on the Microstructure and Properties of Al-Zn-Mg-Cu Alloys

**DOI:** 10.3390/ma15196960

**Published:** 2022-10-07

**Authors:** Qiao Liao, Jianyu Li, Jianping Liu, Shulin Lü, Lu Chen, Wei Guo, Shusen Wu

**Affiliations:** 1 State Key Lab of Materials Processing and Die & Mould Technology, School of Materials Science and Engineering, Huazhong University of Science and Technology, Wuhan 430074, China; 2 Zhejiang Wanfeng Motorcycle Wheel Co., Ltd., Xinchang 312500, China

**Keywords:** Al-5Ti-0.2C refiner, Al-Zn-Mg-Cu alloy, ultrasonic vibration

## Abstract

In this paper, the refinement effect of Al-5Ti-0.2C refiner on Al-Zn-Mg-Cu alloys was first investigated, and then the effects of three forming processes, i.e., Gravity Casting (GC), Squeeze Casting (SC), and Squeeze Casting after Ultrasonic Treatment (UT-SC), on microstructure and properties of Al-Zn-Mg-Cu alloys were studied. The results show that the refining effect of Al-5Ti-0.2C refiner is obvious; first, the average grain size of the alloy decreases and then increases with the increase in Ti content from 0.15 wt.% to 0.3 wt.%. The optimal amount of added Al-5Ti-0.2C is 0.2 wt.% Ti content. The good refining effect is attributed to the formation of TiC particles and Al_3_Ti compounds by the refiner, which can all be the nucleus of solidification. The poor refining effect when the Ti content was more than 0.2 wt.% is due to the formation of coarse Al_3_Ti particles. The results of three forming processes that cast Al-Zn-Mg-Cu alloys under the addition of Al-5Ti-0.2C with 0.2 wt.% Ti content show that the mechanical properties under the UT-SC process are the best; the tensile strength in the as-cast state reaches 367 MPa, and the elongation is 3.84%. The effect of tiny TiC particles in the refiner on the microstructure and properties of Al-Zn-Mg-Cu alloys is also discussed.

## 1. Introduction

Al-Zn-Mg-Cu alloy is an ultra-high-strength aluminum alloy composed of many elements, with high specific strength and stiffness and good processing performance [1]. It is widely used in aircraft, armor plates, mobile bridges, military vehicles, and storage tanks, etc. [2,3]. Since Al-Zn-Mg-Cu alloys always achieve good mechanical properties through heat treatment strengthening, many researchers have explored the effect of Sc, Zr, Ti, and Er on precipitation the kinetics and aging behavior of Al-Zn-Mg-Cu alloys from the aspect of trace elements [4,5,6,7,8,9]. The initial solidified microstructure has a great influence on the subsequent heat treatment results. However, Al-Zn-Mg-Cu alloys contain a great amount of alloying elements, which leads to a severe segregation tendency of the solidified structure and the formation of coarse dendrites and casting defects [10].

Grain refinement is one of the common ways to improve the solidification microstructure and enhance the mechanical properties. In recent decades, Al-Ti-B refiners have been extensively used in the industrial field and are currently recognized as one of the most effective refiners [11]. Many studies have shown that Al-Ti-B refiners indeed have some refining effect, but TiB_2_ particles easily agglomerate, resulting in a smaller quantity of heterogeneous nuclei [12,13]. Moreover, Al-Ti-B master alloys demonstrate more severe Zr poisoning effects compared to Al-Ti-C alloys [14]. Therefore, Al-Ti-C refiner is a favorable option for the refiner of Al-Zn-Mg-Cu alloys. The addition of Al-Ti-C refiner can not only promote heterogeneous nucleation, but also pin crystal boundaries, which is beneficial for the subsequent heat treatment performance. Xiaodong Wu et al. investigated the refinement effect of Al-Ti-C, which depends mainly on the heterogeneous nucleation of TiC particles when Ti content is lower than 0.15 wt.% [15]. Liu Xiangfa et al. found that the grain refining efficiency of Al-Ti-C master alloys largely depends on the surface characteristic, size, and distribution of carbides [16]. Some research indicated that tiny TiC particles could improve the ductility of Al-Cu alloys due to the formation of the finer α-Al dendrite and participates [17]. The refinement effects and mechanical properties of Ti content exceeding 0.15 wt.% for Al-Zn-Mg-Cu alloys still need to be explored.

Although Al-Zn-Mg-Cu alloys are usually DC cast, one of the current development trends is that many 7xxx aluminum alloys are also used to directly cast into parts. In addition to the method of adding refiners, changing the casting processes, for example, ultrasonic treatment–squeeze casting (UT-SC), is also one of the effective ways to promote the mechanical properties of alloys [18,19]. On the one hand, squeeze casting (SC) can eliminate the shrinkage defects of ingots and obtain the material products with dense structure [20]. On the other hand, a faster cooling rate is beneficial for the refinement of α-Al grains. In addition, ultrasonic treatment for the melt can not only promote the solid solution of alloy elements in the matrix, but also improve the segregation defects of alloy elements due to cavitation and acoustic streaming effects. For example, Cheng Li et al. studied the effect of ultrasonic vibration treatment on 7050 alloy with trace element Zr, and found that ultrasonic energy can accelerate the cooling rate of the solidification front, effectively increase the solid solution of alloying elements in α-Al grains, and inhibit Zr segregation [10]. It is also beneficial to improve the distribution of nucleated particles in the matrix and decrease the agglomeration of particles [21]. Jianyu Li et al. applied ultrasonic vibration to nano-SiC particle-reinforced Al-5Cu composites, which improved the distribution of nanoparticles and dispersed the agglomerated particles into separated particles [22]. During the melting process of Al-Ti-C refiner, tiny TiC particles, with a small specific surface area, are easy to agglomerate and sink, resulting in a decline in refining ability. In this study, UT is expected to disperse agglomerated TiC particles in the melt to acquire better refinement effects, reduce the solidification defects of the alloy, and lay the foundation for obtaining a good heat treatment microstructure.

Therefore, in this paper, Al-5Ti-0.2C refiner was added to Al-Zn-Mg-Cu alloy to investigate the effects of more than 0.15 wt.% Ti or the excessive addition of Al-5Ti-0.2C refiner on the microstructures and mechanical properties of alloys. In addition, through changing casting processes, the influence of ultrasonic vibration on the distribution of refining particles and the refinement mechanism of Al-Zn-Mg-Cu alloys was also studied.

## 2. Experimental Methods

**Figure 1:**
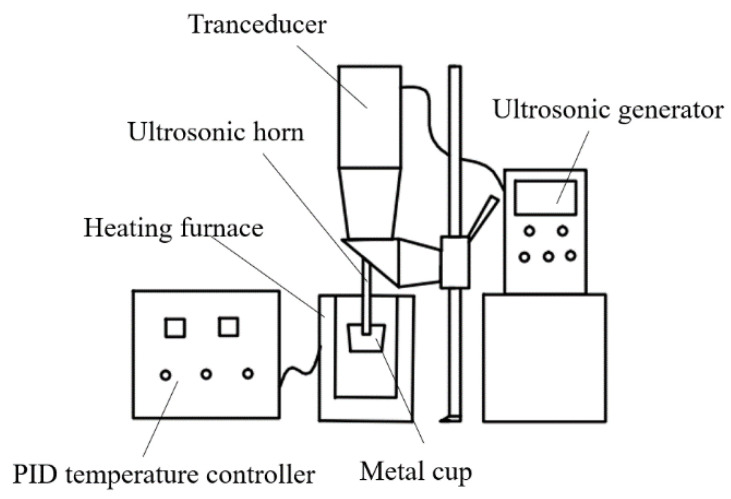
Diagram of ultrasonic vibration treatment equipment.

The raw materials of the samples are composed of pure aluminum, pure magnesium, pure zinc, pure copper, and Al-10Mn (in wt.%, the same in the following), Al-5Cr, and Al-5Ti-0.2C master alloys. Al-5Ti-0.2C master alloys are equivalent to Al-4.2Ti-0.2TiC according to TiC content. The chemical compositions and the casting processes of the samples are presented in Table 1. Gravity casting (GC) was used for the four melts with four different of Ti contents (0.15 wt.%, 0.2 wt.%, 0.25 wt.%, 0.3 wt.%) by adding Al-5Ti-0.2C master alloy. Then, the composition with best performance was selected to obtain SC and UT-SC samples. The materials of the Al-Zn-Mg-Cu alloys were put into a graphite crucible heated by a resistance furnace at 750 °C. After the metals were fully melted, Al-5Ti-0.2C refiner was added to the melt at 720 °C. Argon gas was introduced at 720 °C to degas for 15 min. Taking the UT-SC process as an example, the experiment equipment is shown in Figure 1. The melt was stirred in the graphite crucible before being removed in a ladle.

The treated melt volume was 80 mL. The melt in the ladle was kept warm by the furnace during the ultrasonic time. The ultrasonic horn, made of TC4 alloy, was preheated to 700 °C. The ultrasonic power and frequency were 2 kW and 20 kHz, respectively, and the ultrasonic time was 2 min. Finally, the treated melt was poured into the metal mold in a squeeze machine at 670 °C. The squeeze-casting pressure was 100 MPa, and the temperature of the preheated mold was 200 °C. Finally, cylindrical casting ingots (Φ30 * 110 mm) were obtained.

The samples were taken from the center of the as-cast ingot, ground by sandpapers, polished, and etched with 0.5 vol.% HF solution to obtain a prepared sample. The morphology and phase composition were observed and analyzed by optical microscope (OM) and scanning electron microscope (Nova NanoSEM 450, FEI Ltd., Eindhoven, Netherlands), the source of the SEM signal in this paper was all backscattered electrons). Furthermore, the statistics of each sample’s average grain size were obtained by Image-Pro Plus software (Version 6.0) (five SEM images of one sample were taken to calculate the mean value of an average grain size of each picture). Phase analysis was carried out by a Shimadzu XRD-7000s X-ray diffractometer (Shimadzu Ltd., Tokyo, Japan) with Cu K_α_ radiation at 40 kV, 30 mA. The scanning range varied from 10° to 90° and its speed was 10 °/min. The strengths and elongations at room temperature were tested by a Shimadzu AG-IG100KN (Shimadzu Ltd., Tokyo, Japan) machine.

## 3. Results

### 3.1. Effects of Refiner Contents on Microstructure and Properties of Al-Zn-Mg-Cu Alloys

**Figure 2:**
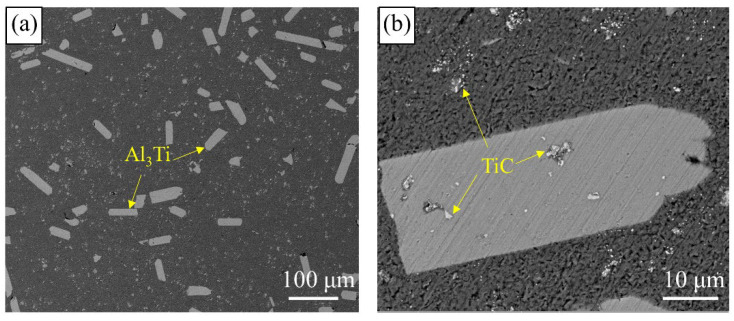
The morphology of grain refiner Al-5Ti-0.2C: (**a**) Overall distribution of Al_3_Ti in Al-5Ti-0.2C; (**b**) The distribution of TiC.

The microstructure of the raw Al-5Ti-0.2C is shown in detail in Figure 2. SEM images of the sample revealed that there are two different phases distributed uniformly in raw Al-5Ti-0.2C refiner. According to EDS analysis, one was strip-shaped Al_3_Ti with a length of about 40 μm. Another was fine granular TiC particles, the size of which was 500 nm to 1.5 μm. Part of the TiC was located in the Al_3_Ti. It was certified that the nucleation location of the Al_3_Ti has a connection with the TiC [23].

**Figure 3:**
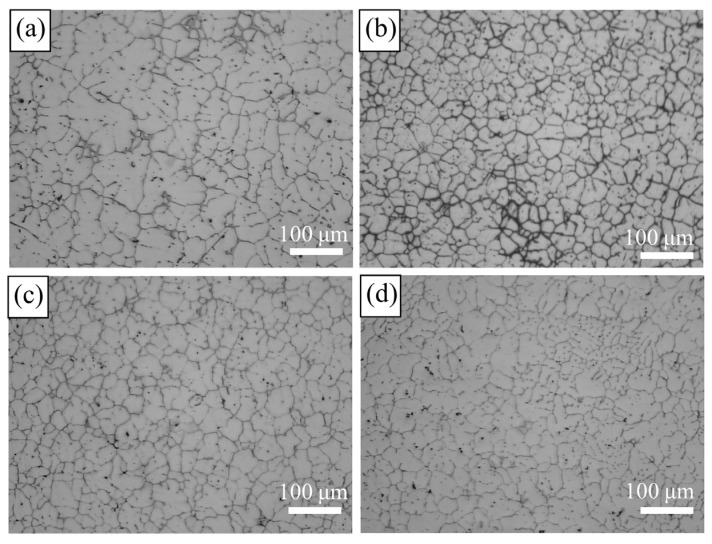
OM (optical microscope) images of alloys with different Ti contents: (**a**) 0.15 wt.%; (**b**) 0.20 wt.%; (**c**) 0.25 wt.%; (**d**) 0.30 wt.%.

The addition of Al-5Ti-0.2C refiner to Al-Zn-Mg-Cu alloys had a remarkable effect on grain size. The metallographic structure of the alloys is shown in Figure 3. With the increase in Ti content, the grain size of Al-Zn-Mg-Cu first decreased and then increased. With the addition of 0.15 wt.% Ti, the grain shape was rose-like and uneven. In addition, the grain boundary was dendrite-like owing to non-equilibrium eutectic solidification on dendrites. As the Ti content increased, the refining effect was more obvious. When the Ti content was 0.2 wt.%, the average grain size was smallest. The shape of the grains became round and equiaxed, and the obvious dendrites disappeared. As the Ti content continued to increase to 0.3 wt.%, it occurred that some grains grew coarser than surrounding grains. Moreover, many black dots, such as second phase, existed in the interior of the grains. The alloy with 0.2 wt.% Ti exhibited the optimal refining effect.

**Figure 4:**
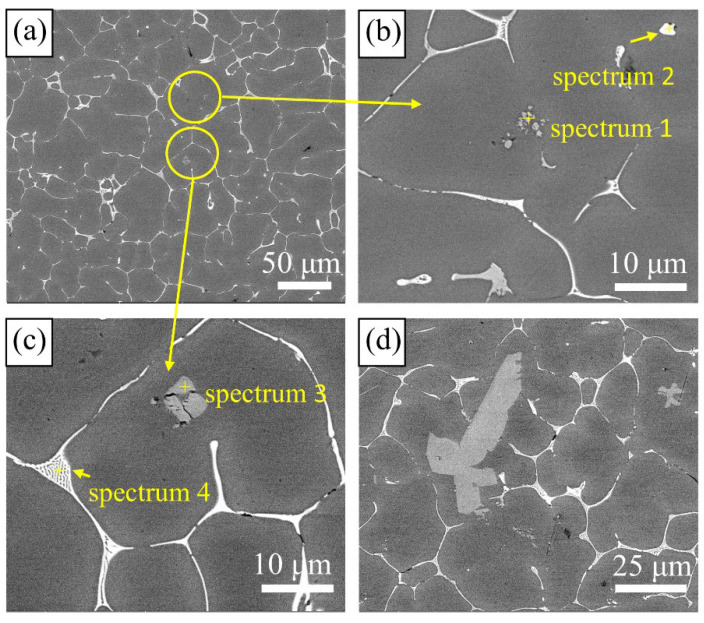
Particle distribution of alloys with different Ti content: (**a**–**c**) 0.20 wt.%; (**d**) 0.30 wt.%.

**Figure 5:**
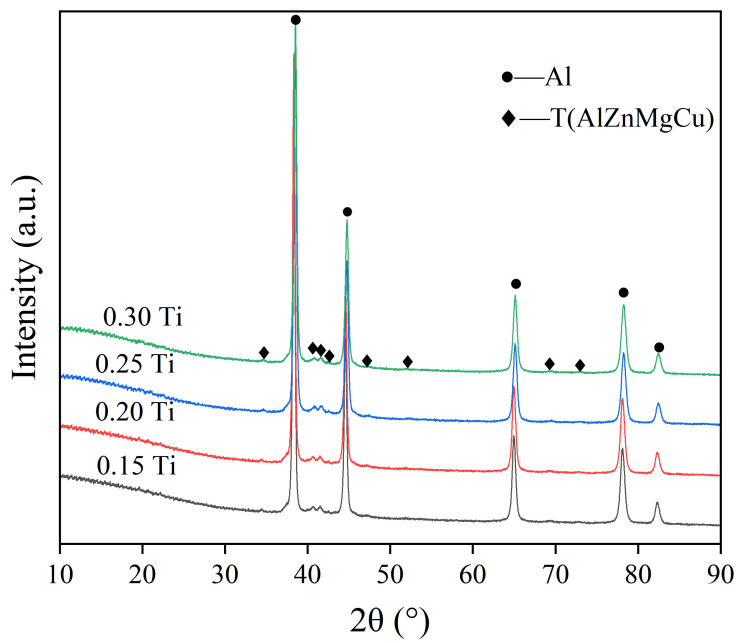
XRD patterns of alloys with different Ti contents.

SEM images showing more specific states of the microstructures and compositions of Al-Zn-Mg-Cu alloys with different Ti contents are provided in Figure 4. EDS results (Table 2) reveal the phase of spectrum 3 was Al_3_Ti compounds; the atomic ratio of Al and Ti was 3:1, and some granular particles with a size of about 500 nm to 1 μm aggregated together (Figure 4b, spectrum 1). These tiny particles were TiC, and they were in the center of the α-Al phase. It was observed the size of coarse Al_3_Ti compound was up to 50 μm in alloys with 0.3 wt.% Ti (see Figure 4d). It was uncovered that Al_3_Ti grew with an increase in Ti content. The composition of the grain boundaries and internal white second phase was still T(AlZnMgCu) phase, which did not change due to the addition of Al-Ti-C refiner (spectrum 4 and 2). The XRD diffraction patterns of the gravity casting samples can also confirm this (see Figure 5). It indicated clearly that, apart from the matrix α-Al phase, the second phase of the sample is mainly T (AlZnMgCu) phase, and the contents of Al_3_Ti and TiC phases were too low to be determined.

**Figure 6:**
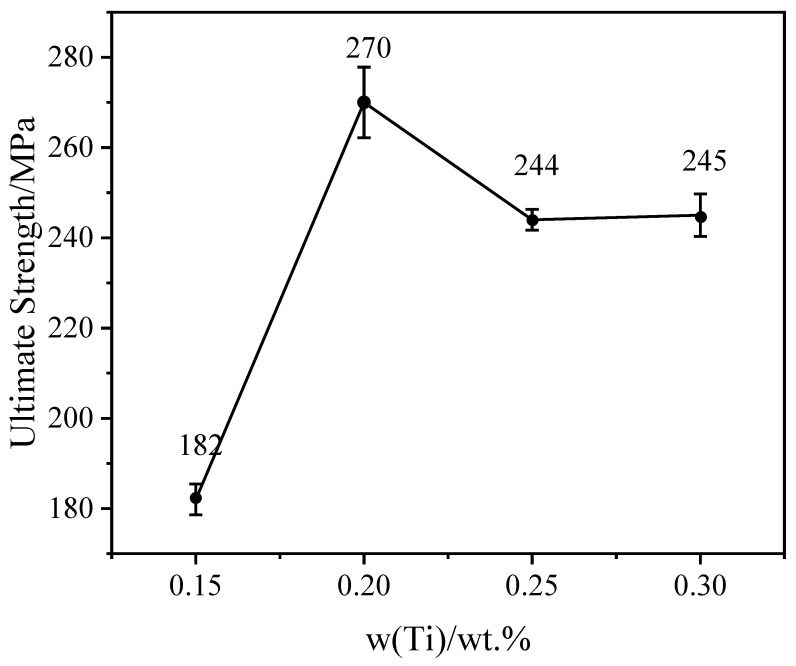
Tensile strength of GC samples with different Ti contents.

Statistical analysis was conducted on the mechanical properties of Al-Zn-Mg-Cu alloys with different degrees of refinement (Figure 6). With the increase in Ti content, the tensile strength of the samples increased at first and then decreased. Among the four additions, the alloy with 0.2 wt.% Ti content had the highest ultimate tensile strength of 270 MPa in the as-cast state, 32% higher than that of alloys with 0.15 wt.% Ti content. When the Ti content is higher than 0.2 wt.%, the tensile strength of the samples started to decrease. Moreover, with the increase in Ti content, the elongation of the samples increased at first and then decreased, and remained stable afterwards, the values of which were 0.31%, 1.00%, 0.52%, and 0.60%, respectively. It showed that the plasticity of Al-Zn-Mg-Cu alloys with Al-Ti-C refiner was related to the Ti concentration.

### 3.2. Effects of Casting Process on Microstructure and Properties of Al-Zn-Mg-Cu Alloys

**Figure 7:**
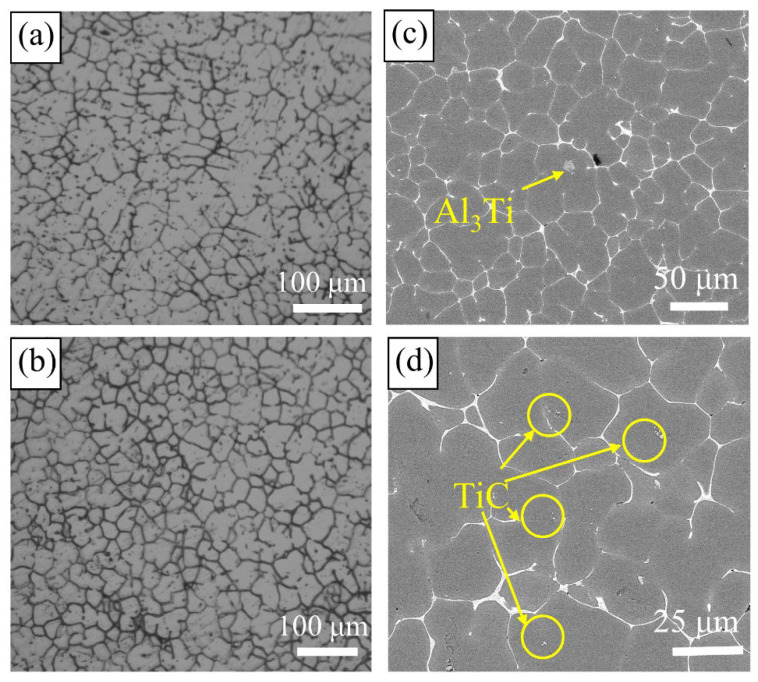
Microstructure of SC samples and UT-SC samples with 0.2 wt.% Ti: (**a**) OM image alloys with SC process; (**b**) OM image with UT-SC process; (**c**,**d**) different magnification of samples with UT-SC process.

SC and UT-SC are believed to improve the plastic properties of alloys remarkably. Figure 7 shows microstructural images of an SC sample and a UT-SC sample. Figure 7a shows that the grains of the SC sample were irregular in shape, with many dendrites, and the grain boundaries were locally coarsened. Figure 7b is the metallographic diagram of the UT-SC samples. The grain shape was relatively round and uniform with the smallest grain size, which had a near-spherical equiaxed shape. Compared with the squeeze-cast sample (Figure 7a), the number of intragranular secondary phases (Figure 7b) was also greatly reduced. Furthermore, it was exhibited that, compared to the SC samples, cavitation and acoustic streaming effects generated by ultrasonic vibration endowed UT-SC samples with more uniform grains.

**Figure 8:**
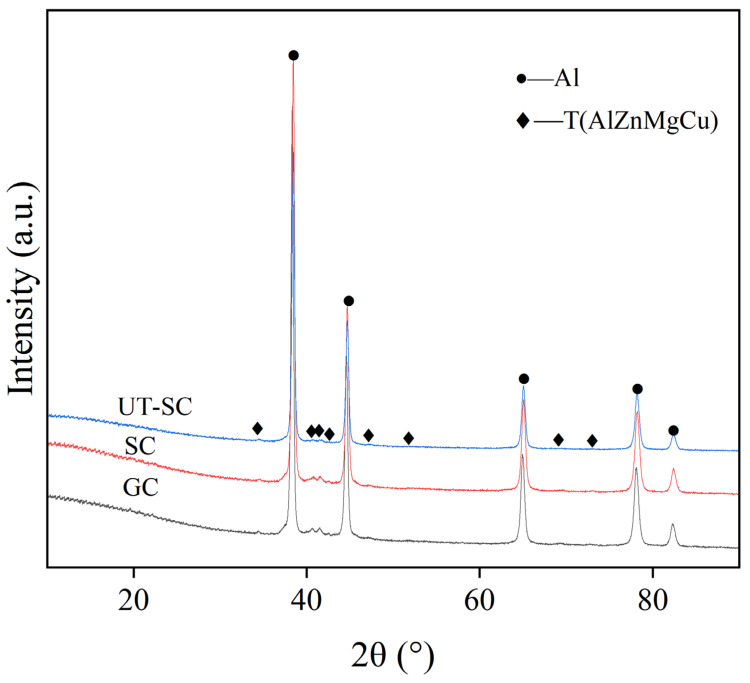
XRD patterns of alloys with different casting processes.

The distribution of TiC particles and Al_3_Ti in the UT-SC samples can be seen in Figure 7c,d. It can be clearly observed that TiC particles were dispersed in each grain (Figure 7d) rather than aggregated together (Figure 4b). The XRD diffraction patterns in Figure 8 illustrate that the main second phases in the SC sample and the UT-SC sample were still the T(AlZnMgCu) phase. Changing the casting process did not change the phase composition. However, the peaks of the T phase in the UT-SC samples became weaker and wider.

**Figure 9:**
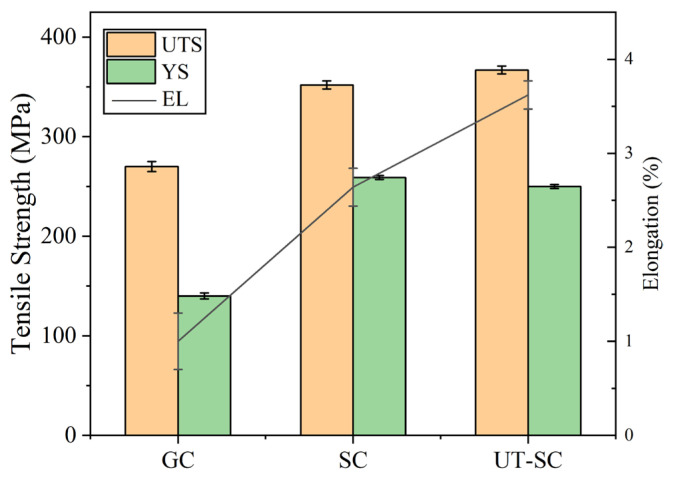
Tensile properties of alloys with different processes in as-cast state.

It was mentioned that the elongations of the GC samples were less than 1.00%. By contrast, elongation of the SC samples with 0.2 wt.% Ti content increased to 2.64% (see Figure 9). After introducing ultrasonic vibration to the melt, the elongation increased to 3.83%. Meanwhile, the tensile strength of the squeeze-cast samples climbed from 270 MPa to 352 MPa, an increase of 32%, and the yield strength increased from 140 MPa to 258 MPa. The tensile strength of the UT-SC sample increased to 367 MPa, which was 38% higher than that of the GC samples.

## 4. Discussion

### 4.1. The Refinement Mechanism of Al-Ti-C Master Alloy

**Figure 10:**
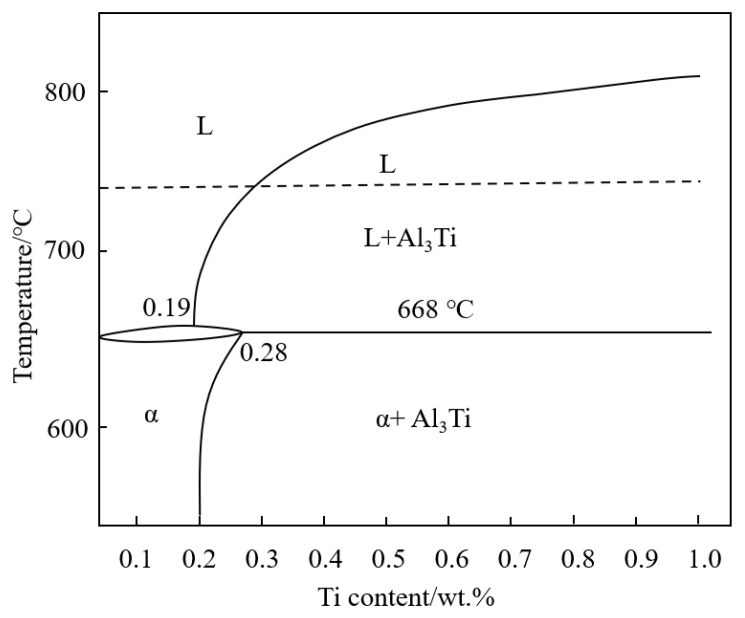
Al-Ti partial phase diagram in the Al-rich side.

At present, there are many explanations for the refinement mechanism of Al-Ti refiner added to Al alloys. In this study, after adding Al-5Ti-0.2C to the metal melt, Ti exists in two forms: TiC particles and Al_3_Ti compounds. The Al_3_Ti compound is unstable and then melts after being added to the melt; TiC particles have a melting point as high as 3100 °C and remain stable particles in the melt. In case of different Ti contents, free Ti has different effects on nucleation situations. Figure 10 shows an Al-Ti partial phase diagram in the Al-rich side [24]. In Figure 10, it is found that: (i) when Ti content is lower than 0.19 wt.%, the liquid solidifies directly into α-Al phase, and at 0.19 wt.% Ti with a peritectic —L+Al_3_Ti→α-Al—there is no primary Al_3_Ti precipitation; (ii) when Ti content is in the range of 0.19 wt.% to 0.28 wt.%, there is primary Al_3_Ti solidification at first, and then a peritectic reaction; and (iii) when Ti content is more than 0.28 wt.%, more primary Al_3_Ti is generated. Therefore, the optimal refinement effect can be obtained with the addition of about 0.2 wt.% Ti, due to a large number of nuclei formed by a peritectic reaction. As the Ti content continued to increase, the size of the grains increased slightly, indicating that the refinement effect on the matrix grains by continuing to add Al-Ti was not obvious. As shown in Figure 4d, when the Ti content is increased to 0.3 wt.% with Al-5Ti-0.2C, obvious coarse Al_3_Ti can be observed. Enzhao Wang et al. proposed a growth model to explain the mechanism of solute-suppressed nucleation and claimed that the release of solidification heat during the process of heterogeneous nucleation could offset the undercooling, even melt the existing nuclei [16]. When the Ti content is higher, the probability of collision and aggregation between particles is higher. When the heterogeneous nucleus Al_3_Ti is formed, solidification heat is released to inhibit the growth of the surrounding heterogeneous nucleus, and remelting may even occur. The excess Ti atoms promote the coarsening and growth of Al_3_Ti and have less effect on grain refinement.

In addition to the nucleation effect of Al_3_Ti, the lattice mismatch between the stable TiC particles and α-Al is 6.87%, which is a semi-coherent interface, resulting in a certain effect on refining the metal grains. According to recent research by Yang et al. on the refinement mechanism of Al-Ti-C, the nucleation interface between the Al matrix and TiC particles is “Al-interface transition layer-TiC”, and the interfacial transition layer is composed of Al atoms and Ti atoms [25]. It is beneficial to reduce the lattice mismatch between the TiC and Al interface. Figure 4b displays the distribution of TiC in the GC sample. TiC particles are distributed inside the sample grains. TiC particles are not moved to the grain boundaries during solidification, indicating that TiC and α-Al have a well-bounded interface. During the solidification of the alloy, the concentration of Ti atoms in the melt is locally too high, and Ti atoms diffuse from a high concentration to low concentration. The free energy of Ti atoms in the melt is higher than that at the interface between Ti atoms and TiC, and Ti atoms aggregate to the surface of TiC particles. Furthermore, the interface transition layer is generated on the surface of TiC particles under the influence of energy fluctuation and increased undercooling. Table 3 shows the difference in the properties of Al-Zn-Mg-Cu alloys with Al-5Ti-0.2C refiner and Al-5Ti refiner (the data came from our previous experiments). It indicates that, compared to alloys with Al-5Ti refiner, alloys with Al-5Ti-0.2C refiner have a significant improvement in ultimate tensile strength. This shows the positive effect of TiC particles to some extent.

### 4.2. The Effect of Casting Processes

The effects of casting process on the micro-scale solidification and overall properties of Al-Zn-Mg-Cu alloys are divided into two parts. Firstly, compared with gravity casting, squeeze casting eliminates the shrinkage porosity defect, and as the pressure increases, the solidification temperature of the melt rises, and the undercooling of the alloy is promoted. In addition, under the action of the pressure, the alloy sample and the mold have no contraction gap, which enhances the cooling conditions. Both can improve the nucleation rate. Secondly, the ultrasonic treatment of the melt before casting uses the acoustic flow effect and cavitation to act on the alloy. On the one hand, because Al-Zn-Mg-Cu contains many metal elements, the segregation tendency is severe. Ultrasonic vibration can promote convection and weaken the segregation. As shown in Figure 8, the diffraction peak of the T phase of the UT-SC sample becomes weak, which reduces the numbers of T phases in the grain boundaries and grains. On the other hand, the refinement effect of Al-Zn-Mg-Cu alloys has a correlation with the distribution state of TiC particles. Tiny TiC particles are inclined to agglomerate and sink in the melt due to their small specific surface area, resulting in a decline in the refinement effect. Thus, a huge amount of energy arising from the ultrasonic vibration, in forms of acoustic streaming and cavitation bubbles, is used to break the agglomeration of TiC particles. In brief, the ultrasonic vibration can promote the diffusion of Ti element in the melt, disperse the agglomeration of TiC (as shown in Figure 7d), increase the number of heterogeneous nuclei, and lessen the grain size, which is beneficial for enhancing the mechanical properties of the alloys [26].

## 5. Conclusions

(1) Addition of Al-Ti-C refiner containing tiny TiC particles has a remarkable effect on the grain refinement of Al-Zn-Mg-Cu alloys. The Ti element exists in the form of TiC particles and Al_3_Ti compounds, both of which can be used as nucleation sites to significantly refine the grains. Among the four added contents, the grain size at 0.2 wt.% Ti addition is smallest.
(2) When the Ti content is 0.2 wt.%, the mechanical properties of the alloys are also optimal. Compared with the initial addition of 0.15 wt.%, the ultimate tensile strength is increased by 32%, and the elongation is increased three-fold. With the addition of 0.3 wt.% Ti content, some coarse Al_3_Ti appeared. Due to the formation of the coarse Al_3_Ti, its solidification heat affects the growth of other Al_3_Ti near the coarse, therefore less effect of grain refinement.
(3) Cavitation and acoustic streaming effects arising from ultrasonic treatment can break the TiC agglomeration, increase more nucleation sites, and refine the grains further. After ultrasonic treatment for the melt with 0.2 wt.% Ti content, the tensile strength and elongation of UT-SC samples is further increased to 367MPa and 3.83%, which are an increase of 36% and 283%, respectively, compared with GC samples.

## Figures and Tables

**Table 1:** Compositions of the alloy samples (in wt.%) and casting processes.

Zn	Mg	Cu	Cr	Mn	Ti	Al	Casting Process
6.43	1.96	2.06	<0.05	<0.1	0.16	Bal.	GC
6.45	1.94	2.07	<0.05	<0.1	0.20	Bal.	
6.44	1.92	2.03	<0.05	<0.1	0.24	Bal.	
6.46	1.95	2.04	<0.05	<0.1	0.31	Bal.	
6.44	1.93	2.03	<0.05	<0.1	0.21	Bal.	SC or UT-SC

**Table 2:** EDS results of phases in Figure 5 (at. %).

Spectrum	Al	Zn	Mg	Cu	Ti	C
1	-	-	-	-	69.62	30.38
2	63.71	11.6	14.71	9.8	-	-
3	74.65	-	-	-	25.35	-
4	78.18	7.74	8.9	5.18	-	-

**Table 3:** The ultimate tensile strength (MPa) of alloy samples with different refiners.

Ti Addition and Casting Process	Al-5Ti Refiner	Al-5Ti-0.2C Refiner
0.15 wt.% Ti + GC	-	182 ± 2
0.20 wt.% Ti + GC	251 ± 3	270 ± 5
0.25 wt.% Ti + GC	-	244 ± 2
0.30 wt.% Ti + GC	-	245 ± 2
0.20 wt.% Ti + SC	326 ± 5	352 ± 4
0.20 wt.% Ti + UT-SC	340 ± 3	367 ± 4

## Data Availability

The raw/processed data required to reproduce these findings cannot be shared at this time due to technical or time limitations.
